# Osteo-Arthritis

**Published:** 1889-09

**Authors:** 


*Osteo-arthritis.* By JOHN KENT SPENDER, M.D. Lond. London: H. K. LEWIS. 1889.

This essay directs attention to the early symptoms and the early treatment of rheumatoid arthritis. From an analysis of more than a thousand cases of those who visit Bath for the sake of the "water cure," the author picks out certain symptoms as especially connoting osteo-arthritis; and of these he directs especial attention to "increased velocity and tension of the heart's action." He states that "a steady pulse of much tension, varying from 90 to 110, is tolerably common. The pulse quickens synchronously with the earliest objective signs of osteo-arthritis." We are sorry that no sphygmographic tracings are given, and that the statement with regard to high tension is therefore not demonstrated. The point is one of much interest, inasmuch as a quick pulse is commonly one of low tension. We shall hope for further evidence on this point: such a persistent increase of tension as is described would be likely to lead to cardiac hypertrophy and valvular defect, which we know to be the exception in this disease.

Another symptom of much value in diagnosis is a "disturbance in the chromatogenous function of the skin. Concentrated as patches more or less large, the pigmentation assumes many hues, and affects many parts of the body. Across the forehead it often runs as a light bronze smear, or like a patch of chloasma. . . . Without a question being put, people often voluntarily say that the yellowness and the spots began at the same time as the rheumatoidal symptoms." The author describes the various forms of pigmentation, and he appears to rely on these for diagnosis to an extent which is not usually appreciated.

Numerous illustrations of vaso-motorial and neural symptoms conclude the chapter on diagnosis.

The remaining chapter is a powerful appeal for early treatment, in opposition to the usual method of doing little. He pleads "that the proper treatment of osteo-arthritis consists in seizing the right threads at the very beginning, and in holding them with a fighting attitude to the very end." The whole chapter is full of practical experience, and holds out much hope to the patient for whom little expectation of benefit from treatment is commonly entertained. We will not spoil it by abstracting any portion: it should be read as its author gives it, and if acted npon seems likely to give satisfaction both to doctor and patient.

